# A comparative simulation analysis of distributed power flow controller performance on transmission system enhancement

**DOI:** 10.1038/s41598-026-48190-2

**Published:** 2026-04-10

**Authors:** Abhishek Vashistha, Dharmbir Prasad, Pankaj Kumar

**Affiliations:** 1 Department of Electrical and Electronics Engineering, SRM Institute of Science and Technology, Delhi NCR Campus, Modinagar, Ghaziabad, Uttar Pradesh 201204 India; 2 Manipal Institute of Technology, Manipal Academy of Higher Education, Manipal, India

**Keywords:** DPFC, UPFC, Motionless compensation, THD, Power quality, Reactive power, Active power, Energy science and technology, Engineering

## Abstract

Globally all power utilities are striving to cater to the ever-increasing demand for electrical energy by either adding new transmission lines or by optimizing the already existing ones with an objective of transmitting higher degrees of power. The construction of new transmission infrastructure, however, is limited by higher costs and regulatory hurdles. Also, the flow of power in transmission networks oftentimes follows very undesirable pathways and system stability is heavily dependent on voltage changes between lines. A successful answer to these problems is the intelligent regulation of power flow in transmission network. This study provides high-level operational evaluation of Flexible Alternating Current Transmission System (FACTS) strategies, namely Unified Power Flow Controller (UPFC) and Distributed Power Flow Controller (DPFC), which are analysed through their active power exchange characteristics. The UPFC has a common DC link among its shunt-series converters. On the other hand, DPFC does not require a common DC-link and instead replaces each of three‑phase series converters with multiple single‑phase distributed converters which are placed along the transmission line. MATLAB/Simulink is used for modelling and simulation of UPFC and DPFC operating behaviours to observe their performance in controlling power flow with respect to Total Harmonic Distortion (THD). It can be seen from the load flow simulation results, under normal operation condition DPFC transfer larger amount of the active power (i.e., 0.301 MW vs. 0.290 MW) with lower voltage THD (i.e., 9.04% vs. 15.26%) than UPFC. In a three-phase faults scenario, the DPFC continues to carry out better performance with 0.280 MW active power (i.e., UPFC: 0.250 MW), 0.072 MVAR reactive power (i.e., UPFC: 0.058 MVAR) and minimized voltage THD. These findings show that the DPFC outperforms all other FACTS devices in terms of voltage stability and power transfer capacity, confirming its increased reliability for applications involving transmission systems.

## Introduction

Power quality has become one of the top issues regarding electric power utilities in recent years. Power quality suggests to the extent of deviation in transmission and use of electrical energy, affecting the performance of electrical equipment. Medium voltage power systems are prone to voltage disturbances, most of which are manifested in the form of voltage sag; this is one of the main causes of poor power quality. This is generally caused by a connection of heavy loads like large induction motors, industrial boilers connecting quickly. Dynamic voltage restorers (DVRs) have been extensively used to mitigate voltage sags by compensating sudden changes in the system impedance. Additionally, equipment like UPFC and Static Synchronous Compensator (STATCOM) is applied to improve voltage quality at the buses of transmission and distribution. In this paper, the Dynamic Power Flow Controller (DPFC), an active FACTS device, is introduced to solve power and current waveform distortions in a second time frame to improve power quality. The DPFC, which has been proposed initially as a derivation of same UPFC structure with an original one shunt converter actually connected on the transmission line and one or multiple independent single‑phase series converters attached in another. The DPFC has similar functions to UPFC, providing the possibility of controlling parameters of transmission like line impedance, transmission angle and magnitude at bus voltage without a conventional DC link. FACTS technology is intrinsically associated with the evolution of smart grid infrastructure. These devices are essential to enhancing operation, reliability and flexibility of modern power systems. FACTS technologies play a crucial role in intelligent grid operation by enabling dynamic control of power flow, voltage stability, and reactive power compensation. In present era, real-time solutions for voltage regulation^1^, power oscillation damping, and congestion management are increasingly being implemented deploying devices such as STATCOM, SVC (Static Var Compensator) and UPFC.

The different FACTS devices are compared on their operating principles and applications. Only dynamic reactive (DVAR) support and voltage regulation capability can be provided by SVC and STATCOM, respectively; in this context, the latter presents some benefits such faster response time and better performance at low ‑V. As one of the most flexible FACTS devices, UPFC can control voltage magnitude, phase angle and line impedance in real time^2^. MATLAB‑based modelling and simulation for voltage and power management were presented in^3^, while series compensation methods suitable for enhancing power system performance were evaluated and selected in^4^ by Madhuranthaka and Manohar (2016). Power system stability analysis under various operating scenarios is one of the important factors for maintaining secure and uninterrupted operation of the systems^5^. With rising load variability, corrective measures such as the consumer end reactive power compensation have become important to incorporate. Mobile reactive power compensators have flexibility and can be deployed rapidly at points with voltage shortage^6^. Static capacitors are used for VAR regulation in hybrid power systems to procure least-cost real time voltage profiles that would be advantageous, especially in day ahead electricity markets^7^.

As a result, the STATCOM become a fast‑acting FACTS device to provide or absorb reactive current and control voltage at point of common coupling. The STATCOM works similarly to a voltage-source converter, capable of producing or absorbing reactive power as demand dictates to support power quality and grid stability^8–10^. On the other hand, grid‑connected inverters with reactive power capabilities can add extra reactive power requirements because of their filter circuits^11^. Fixed capacitors are a cheap option for reactive power compensation; however, they’re not flexible under dynamic operating conditions^12^. In^13^, comparative studies of FACTS devices have been presented to achieve reliable power flow control, thereby improving overall system controllability. Reactive power compensation has become progressively important, with growing penetration of distributed generation and renewable energy sources^14^. Téllez et al. The optimal sizing and placement of reactive power compensators based on voltage stability indexes (VSIs) like Fast VSI (FVSI), Line Quality Factor (LQF) and VSI^15^ with some iterating method was proposed in [2018]. Voltage stability and proper power flow controller are important for a safe operation of power systems. When active and reactive power flow is not balanced or operating unit cannot afford sufficient reactive power support, then system instability or malfunctioning may occur^16^. FACTS device can be arranged in either series or shunt configurations, with each configuration having its own pros and cons. The FACTS devices of series type are primarily applied to control power flow, damping power oscillations and current regulation while shunt FACTS devices are commonly cast-off for voltage regulation and reactive power compensation. However, series compensation alone often needs system‑specific approaches to handle dynamic load changes and fault scenarios. As such, a coordinated arrangement of series and shunt FACTS controllers is proposed for realizing better power flow control and voltage stability^17,18^.

Note that capacitor banks are passive devices, so do not have the ability of adjusting voltage in dynamic system situations. Therefore, SVCs and STATCOM are favoured to provide real-time reactive power support^19^. The third‑generation FACTS device, STATCOM has gained a great deal of attention due to the limitation of traditional compensation methods. The advantages of D-STATCOM are its higher response time, compatibility with energy storing devices such as batteries and fuel cells, better low‑voltage characteristics, steady reactive current provision, and greater reliability because there are no moving parts^20^. The PSCAD/EMTDC simulation of a combined series‑shunt compensator for power quality enhancement was described by Hannan and Mohamed (2005)^21^. Divan et al. (2004) suggested a distributed static series compensator arrangement for active power flow control in existing transmission lines^22^. Pereira et al. (2021) developed a similar idea for distributed active power flow control^23^. DPFC operation in the presence of shunt converter failure conditions was investigated by Yuan (2009)^24^, and then Liu 1st proposed DPFC to be as a new FACTS device (Yuan 2010)^25^. Barakati et al. (2011) investigated the use of a dynamic voltage restorer (DVR) to compensate for voltage sag and swell with an asymmetric cascaded multicell converter^26^. The extensive use of FACTS controllers in power transmission and distribution systems is discussed by Padiyar^27^. The relationship between DPFC and power improvement was studied in a subsequent study by Jamshidi (2012) who used the synchronous reference frame technique^28^. The design and operating principles of Distributed Power Flow Controller were presented in^29^ by Reddy and Tech (2012) and active power control of transmission lines through DPFC concept was analysed by Gaigowal and Renge (2013)^30^. The practical implementation of this work is as followings:

- *Removal of the shared DC link:* The main difference is that there is no longer a high‑voltage (hv) dc link connecting shunt converter and series converter of any UPFC. This elimination is critical to prevent expensive high‑voltage isolation between series converters and shunt converter.
- *Series converter distribution (D‑FACTS concept):* Unlike a single large three‑phase converter, DPFC utilizes several small independent series converters that are dispersed along transmission line and connected in series.
- *Active power exchange method:* In this approach as the DC link had been eliminated, the DPFC series converters transfer their active power to the shunt converter by means of a transmission line with incorporated third ‑ harmonics.
- *High-pass filter requirement:* Due to the third-harmonic catching power, a high-pass filter should be shunt-connected to line to confine harmonic components and inhibit them from penetrating into grid.

Adopting small‑scale single‑phase converters help to reduce the overall rating loss in power electronics components. Further, since series converters are floating relating to ground, it removes high‑voltage insulation requirements across all phases—a cost reducer. One series converter failure does not break the overall performance, so reliability and redundancy are improved compared to distributed structure. In addition, the fault in one converter has less effect on controller because of the distribution features of series converters, which results in good voltage sag recovery and power oscillation damping performances under fault conditions. With their small dimensions, the distributed converters can be installed directly on present transmission line towers. The present study provides a detailed systematic comparison of UPFC and DPFC in dynamic loading environment and fault scenarios. This study provides a novel perspective on the assessment of power quality indices, as it integrates improvements in factors such as voltage profile, active and reactive power, total harmonic distortion for voltage and current from detectable districts.

While the prior works have focused primarily on steady‑state analysis or individual performance metrics, this study explores the transient behaviour of both controllers during rapid load transients and fault conditions using a comprehensive MATLAB/Simulink model. The experimental findings also fully validate the DPFC’s superior harmonic suppression ability and power transmission capability. This work has the following main contributions:

- Improved power quality results in better system performance.
- Better power factor leading to better system efficiency with lesser losses in line.

None of the reviewed literature has a comparison between performance indices UPFC vs DPFC, which is what this study will provide.

*Paper layout:* The remainder of this paper is organized as follows: Section "Background of UPFC and DPFC technology" gives a brief overview and operating principles of UPFC and DPFC in FACTS technology. In Section "Comparisons between UPFC and DPFC", the following parameters are compared with their representatives. Section "System Configuration and problem identification" presents a detailed description of the system configuration, modelling in MATLAB/Simulink and problem formulation. Simulation results for normal and load variation with three phase fault conditions are presented in Section "Results and discussion" along with power transfer, voltage profiles and THD comparisons. The Section "Conclusion", thus concludes with major findings and advantages of DPFC for contemporary transmission systems.

## Background of UPFC and DPFC technology

### Concept of UPFC

The UPFC is the third generation of FACTS device. Specifically, it is used to control voltage magnitude, phase angle and active & reactive power in transmission lines. Control of three critical parameters in a power system such as line impedance, bus voltage and phase angle at same time and independently are an important feature of UPFC. The UPFC now is one of the most essential devices designed so far, since they are able to react relatively fast and provide efficient reactive power compensation mainly favourable for high voltage transmission (HVDC) system. In a UPFC, voltage injected into the line is controlled by a series voltage-source, which feeds a controllable three-phase voltage having variable magnitude and phase angle. The injected voltage allows active and reactive power to be controlled dynamically. The active power flow among DC link and series inverter, while a shunt inverter is used to keep a continual DC-link voltage by providing or absorbing the necessary active power. This coordination maintains the total active power extracted from the transmission line by UPFC is equal to that lost by the inverters and terminal transformers. The shunt inverter does voltage control at point of common coupling exchanging required reactive power with transmission line. Furthermore, if the DC link gets decoupled, both series and shunt VSC may also undergo independent functioning. In this mode, the shunt inverter operates as a STATCOM and is able to inject or absorb reactive power causing the voltage magnitude of its connection point to change.

**Fig. 1:**
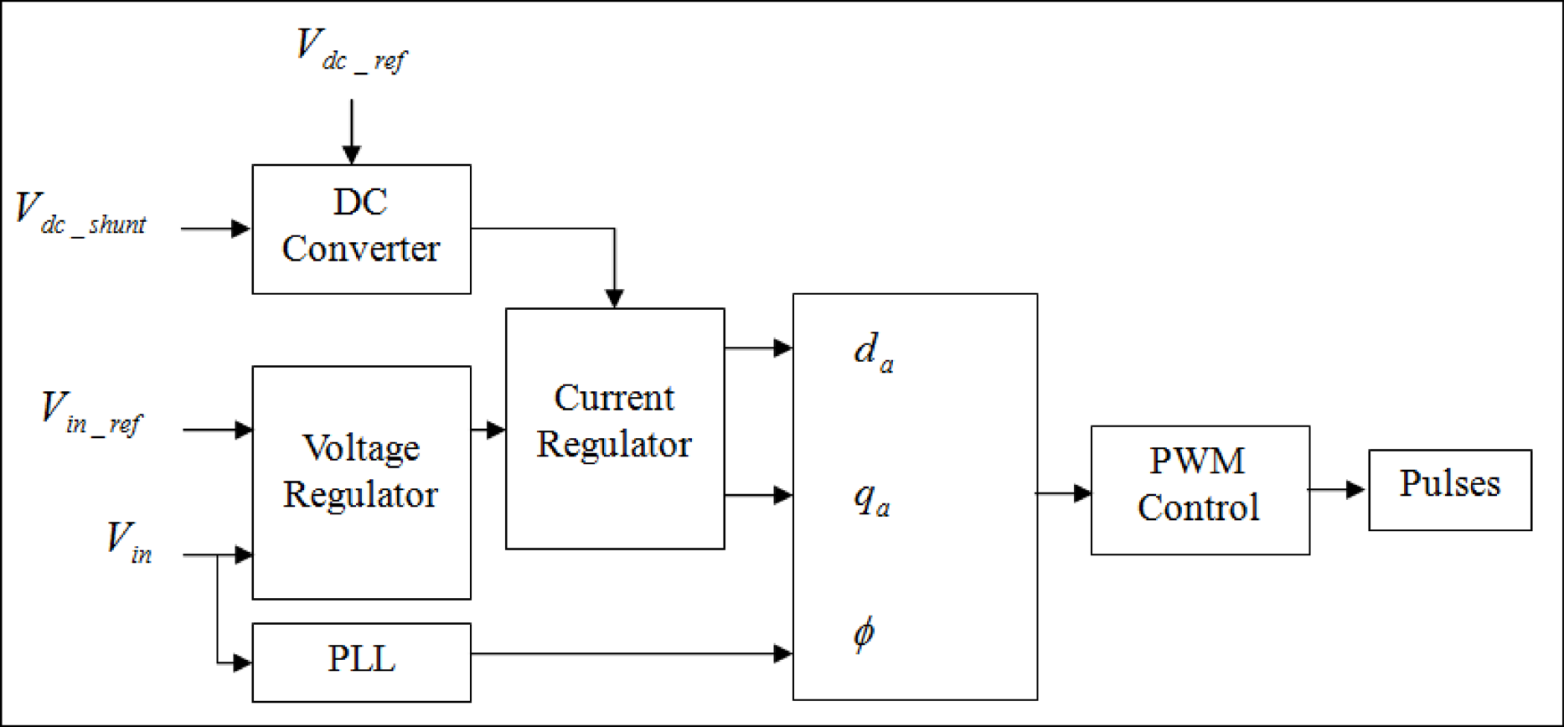
Functional block diagram of UPFC shunt controller.

**Fig. 2:**
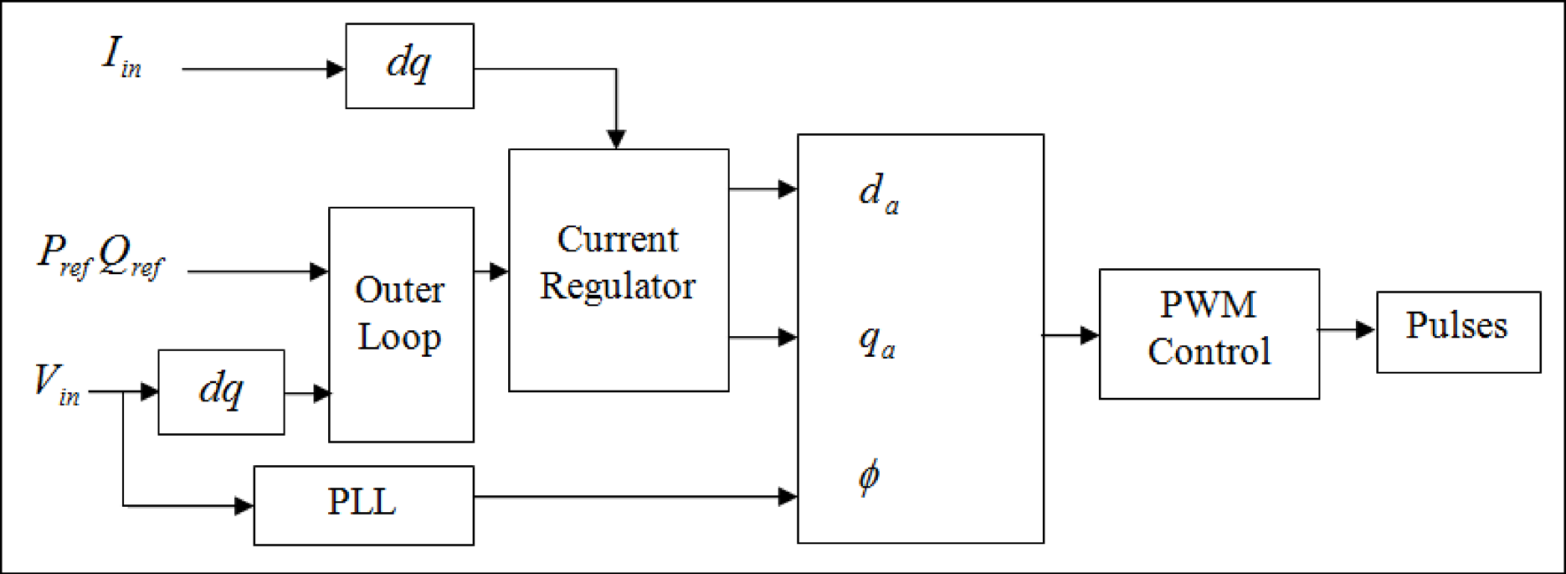
Functional block diagram of UPFC series controller.

As depicted in Fig. 1, the shunt control block diagram shows that the charge controller and maintenance of capacity to preserve a fixed DC-link capacitor voltage are responsibility of the shunt controller. This process allows the series converter to optimize its performance and consequently, regulate the power flow as well as voltage profile more efficiently. As shown in Fig. 2, while series converter injects active and reactive power into grid by using DC-link voltage.

This controller approach only takes dual instance of values of *P*_*ref*_ and *Q*_*ref*_, also equivalences *P*_ref_ and *Q*_*ref*_ through *V*_*d, grid*_ to derive *I* and *I*_q, *ref*_ as stated via (1) and (2)^14^.

1 $${I}_{d,ref}=\frac{\left({V}_{d}{P}_{ref}+{V}_{q}{Q}_{ref}\right)}{\sqrt{{\left({V}_{d}\right)}^{2}+{\left({V}_{q}\right)}^{2}}}$$

2 $${I}_{q,ref}=\frac{\left({V}_{d}{P}_{ref}+{V}_{q}{Q}_{ref}\right)}{\sqrt{{\left({V}_{d}\right)}^{2}-{\left({V}_{q}\right)}^{2}}}$$

### Concept of DPFC

The concept of UPFC helps in improving the power flow control and stabilizes voltage of transmission systems. But DPFC works without a common DC link among shunt and series converters as in case of UPFC. Instead, it utilizes a distributed FACTS architecture, where the traditional three-phase series converter is break into several single-phase series converters placed at various points along the transmission line. In fact, the DPFC can still adjust active and reactive power flow at dc port with composite functioning of one shunt converter along with a number of series converters. The series converters operate independently and capable to manage its individual DC capacitor to keep desired DC voltage. The UPFC directly connects shunt and series converters over a common DC link, while DPFC removes this physical connection with equivalent power flow control abilities. Subsequent, DPFC uses non-sinusoidal voltage and current waveforms that can be demonstrated as superposition of sine parts at a collection of frequencies concerning Fourier evaluation as expressed in Eq. (3)^15^:

3 $$P=\sum_{i=1}^{\infty }{V}_{i}{I}_{i}cos{\emptyset}_{i}$$

The active power in a power system is multiplication of voltage and current. The active power associated with each harmonic frequency is independent as integral of two signals with dissimilar frequencies over one full fundamental cycle equals zero. This relationship is defined in Eq. (3), where, ${V}_{i}$ and ${I}_{i}$ represent voltage and current of ${i}^{th}$ harmonic component, respectively, while, ${\varphi }_{i}$ indicates angle of phase between them. As a result, a converter can receive active power at one frequency and broadcast it at another, enabling dynamic power interaction modes.

**Fig. 3:**
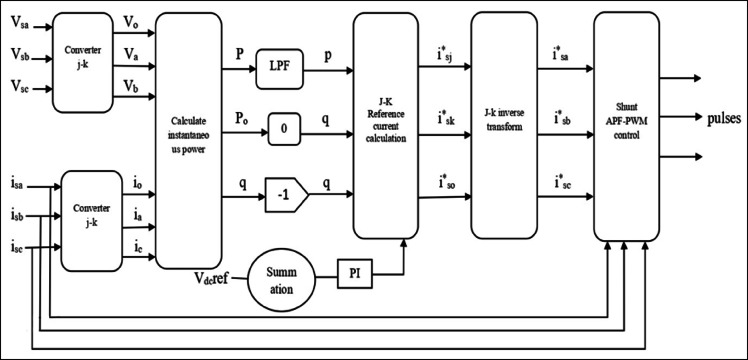
Functional block diagram of DPFC shunt controller.

In DPFC system, the shunt converter pulls active power from grid at fundamental frequency to ensure the operation of system and redistributes it in designated harmonic frequencies. This technique allows the harmonic currents to circulate inside transmission system. The third harmonic components of each phase are equal in a three-phase system, which means that they belong to the class of zero‑sequence harmonics. These zero‑sequence currents circulate in a closed loop through the shunt converter, high‑pass filters and ground. In the case of meshed transmission networks, a grounded star-delta transformer as a common model is normally used for the flow of harmonic current. This configuration offers a way for zero‑sequence harmonics, such as third, sixth and ninth order harmonics so that active power can be shared among converters. Compared to the UPFC, as it uses a number of separate small single‑phase series converters making it more cost-effective, reliable and flexible. In addition, the dispersed configuration of series converters improves system performance and controllability and also minimizes harmonic distortion. The overall DC/DC topology of the DPFC is shown, and it contains of a three-phase shunt converter with a bank of single-phase series converters for voltage regulation and power flow control support. The shunt control of one three‑phase and two single‑phase converters in a back-to-back arrangement is shown in Fig. 3. The shunt converter consumes active power at a fundamental frequency, ensures regulation of DC capacitor voltage, and injects third harmonic current into a neutral conductor of Y-connected transformer.

**Fig. 4:**
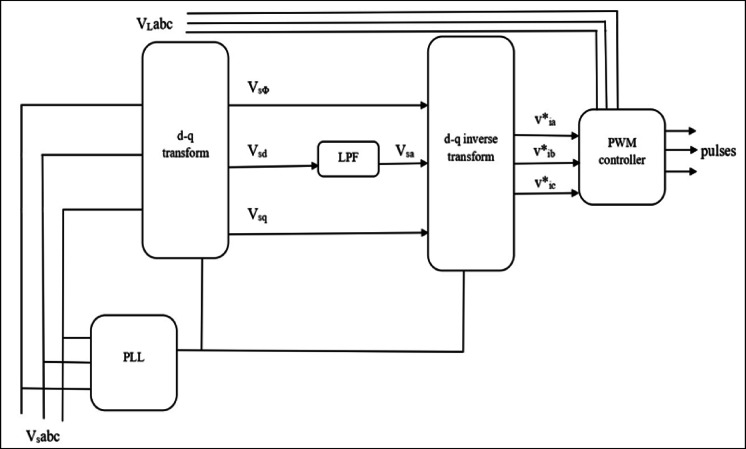
Functional block model of DPFC series controller.

The series control block diagram (shown in Fig. 4), regulates each single‑phase series converter independently in the transmission line. A d‑q control system where the controller gets inputs like capacitor voltage, line current and reference series voltage which are converted into d‑q reference frame for appropriate control actions.

## Comparisons between UPFC and DPFC

Table 1 summarizes the comparison of Unified Power Flow Controller (UPFC) and Distributed Power Flow Controller (DPFC) in terms of different operating and structural parameters.

**Table 1:** Performance comparison of UPFC with DPFC.

Specifications	UPFC device	DPFC device
Controller ability	Low	High
Operative dependability	Low	High
Noise problematic	Noisy	Less noisy
Electrical proficiency	Less effectual	Very effectual
Converter	Two numbers of three-phase	One single shunt and multiple independent series
DC linkage	Available	Eliminated
Power superiority and stability	Average	High
Price	Costly due to three-phase converters ratings	Less costly due to single-phase converters rating
Harmonic problematic	Reduced	Effectively reduced
Fault feedback	High	Very high
Load variation	Less stable for stability	Highly stable for stability

The aforementioned objectives are common goals of the UPFC and DPFC with respect to FACTS, i.e., voltage stability improvement, as well as controlling profitable active and reactive power flow in transmission lines. While goals of both are common, their principles of operations are decidedly different. By applying third‑harmonic frequency components, the DPFC simplifies active power exchange among its shunt and series converters, which reduces complexities in common DC link. In order to maintain each series converter as a redundancy function, while managing safe active and reactive power compensation of associated transmission line. Moreover, the DPFC also uses the transmission line as a medium to transfer third‑harmonic power between the distributed series converters and connected transformers.

## System configuration and problem identification

**Fig. 5:**
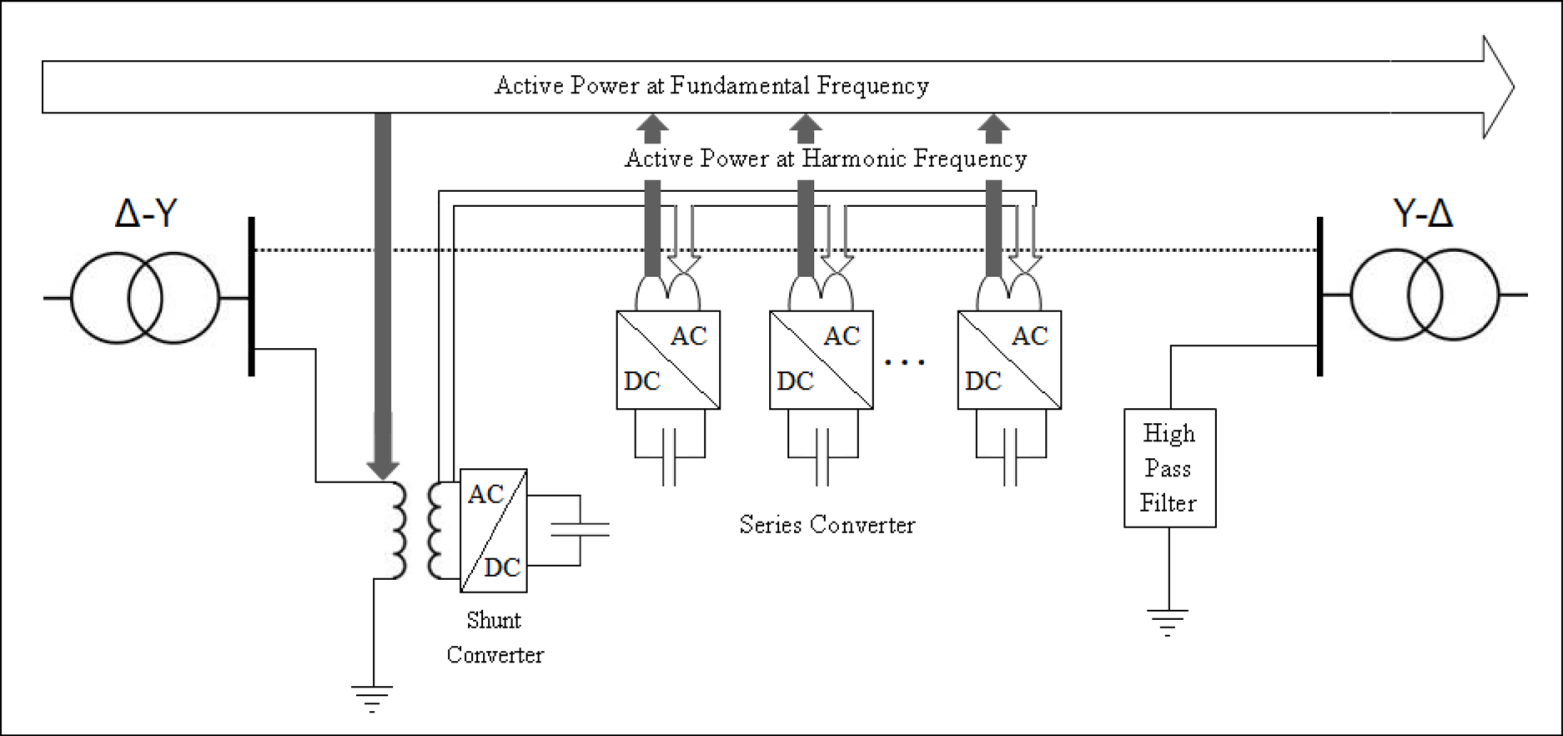
Active power flow in DPFC connected system.

The DPFC structure for the active power exchange is shown in Fig. 5. This block diagram is used to develop a MATLAB/Simulink model to simulate and analyse voltage, THD, and active and reactive power performance. It consists of a shunt converter that absorbs active power from grid at fundamental frequency and feeds third‑harmonic power back to transmission line. The series converter takes third‑harmonic active power and puts fundamental frequency active power into the grid. This operation is referred to here as a “give‑and‑take” mechanism: A third harmonic active power is supplied to the grid by DPFC as a positive (actual) value, while the same amount of the fundamental frequency active power is absorbed from it. High-frequency components not only lead to signal degradation but also affect proper operation of the power supply and therefore, filters are implemented at the AC input in order to suppress unwanted high‑frequency components as well as at the power supply output for voltage ripple reduction.

Compared to the UPFC, the significant benefit that DPFC is a consistent presence of no huge DC link and instead it uses third‑harmonic currents for active power exchanges. Figure 5 depicts the power flow mechanism of the DPFC, where active power flows at fundamental frequency (i.e., 50 Hz) through transmission line and other active power is exchanged at harmonic frequencies. Here is how this analysis is done. Show in the Eqs. (4)-(5)^17,18^:

4 $${V}_{p}={I}_{1}{R}_{1}+{L}_{1}\frac{di}{dt}+{e}_{1}$$

5 $${V}_{i}={I}_{2}{R}_{2}+{L}_{2}\frac{di}{dt}+{e}_{2}$$

The required reactive power and capacitance value is computed using by (6)^17,18^,

6 $${Q}_{s}=\frac{{V}_{2}}{{X}_{c}}$$

In recent years, power quality has remained primary concern for electric utilities. Electrical power is commonly classified into two components: active power and reactive power. Active power performs useful work in an AC circuit, such as driving mechanical loads or generating heat. Reactive power, on the other hand, does not perform useful work but generates the electric and magnetic fields necessary for the operation of inductive and capacitive components. Excessive reactive power leads to higher losses, reduced system efficiency, and a lower power factor, preventing the full utilization of total power. Power quality is a term used in the electrical power supply industry to describe the general condition of the supplied electric power. As a FACTS device, the DPFC is proved to be efficient in doing away with reactive power influence. The DPFC improves overall power quality by compensating reactive power, and also alleviating distortions in voltage and current waveform.

## Results and discussion

The UPFC and the DPFC topologies were simulated by MATLAB/Simulink. In the subsequent subsections, we present and discuss the simulation results.

### Simulation result of UPFC system

**Fig. 6:**
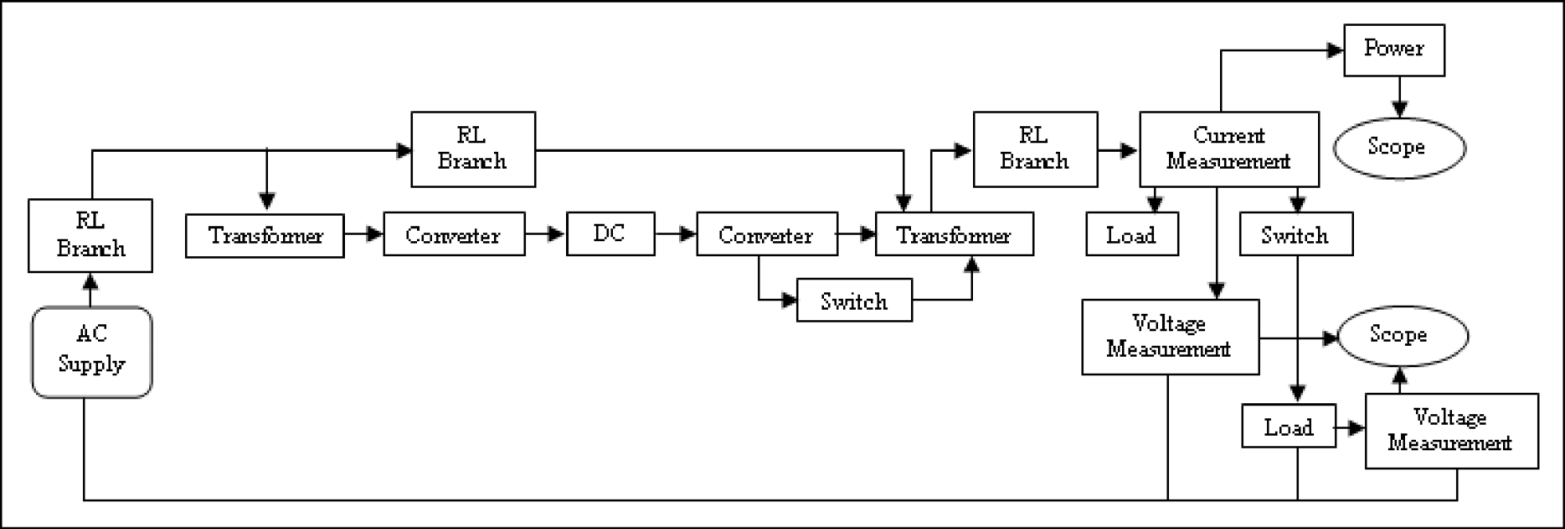
Line model of UPFC structure.

MATLAB/Simulink Model of a UPFC in dual-bus System is illustrated in Fig. 6. The subsystem comprises STATCOM and Static Synchronous Series Compensator (SSSC) where SSSC injects compensating voltage at mid-point of transmission line.

**Fig. 7:**
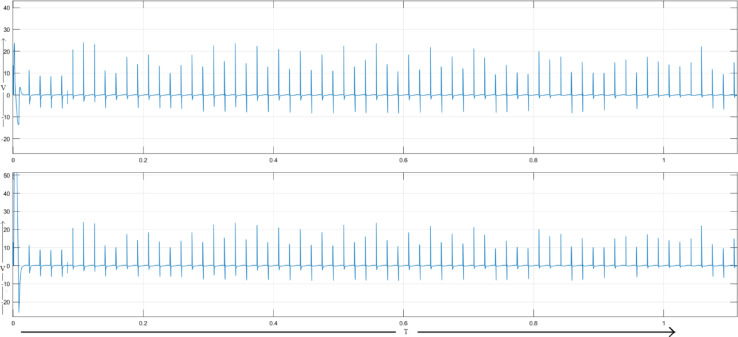
Voltage across load 1 and load 2 for UPFC system.

A load disturbance is initiated at 0.3 s when another load in parallel with the original one has been added. As seen, this increase in voltage drop across the line resistance causes a fall in voltages at Load1 and Load2. As shown in Fig. 7, the UPFC injects a compensating voltage to restore the voltages to their nominal values.

**Fig. 8:**
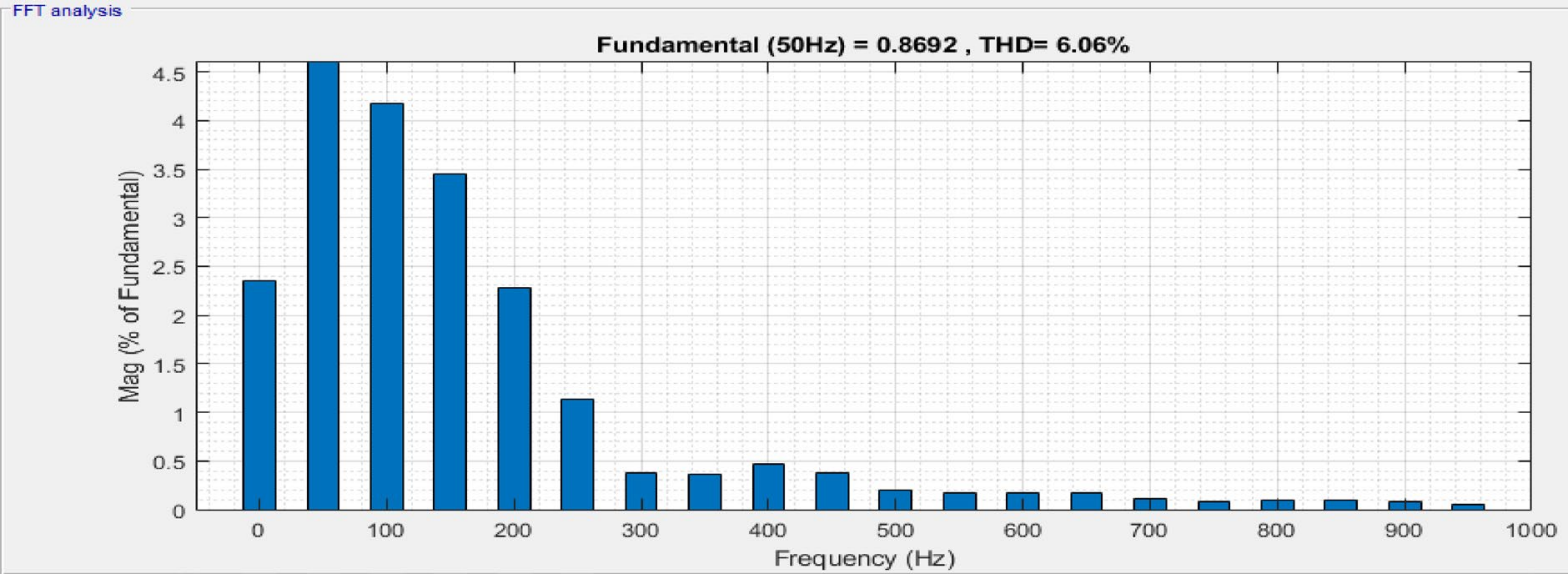
Frequency band for load current of UPFC system.

**Fig. 9:**
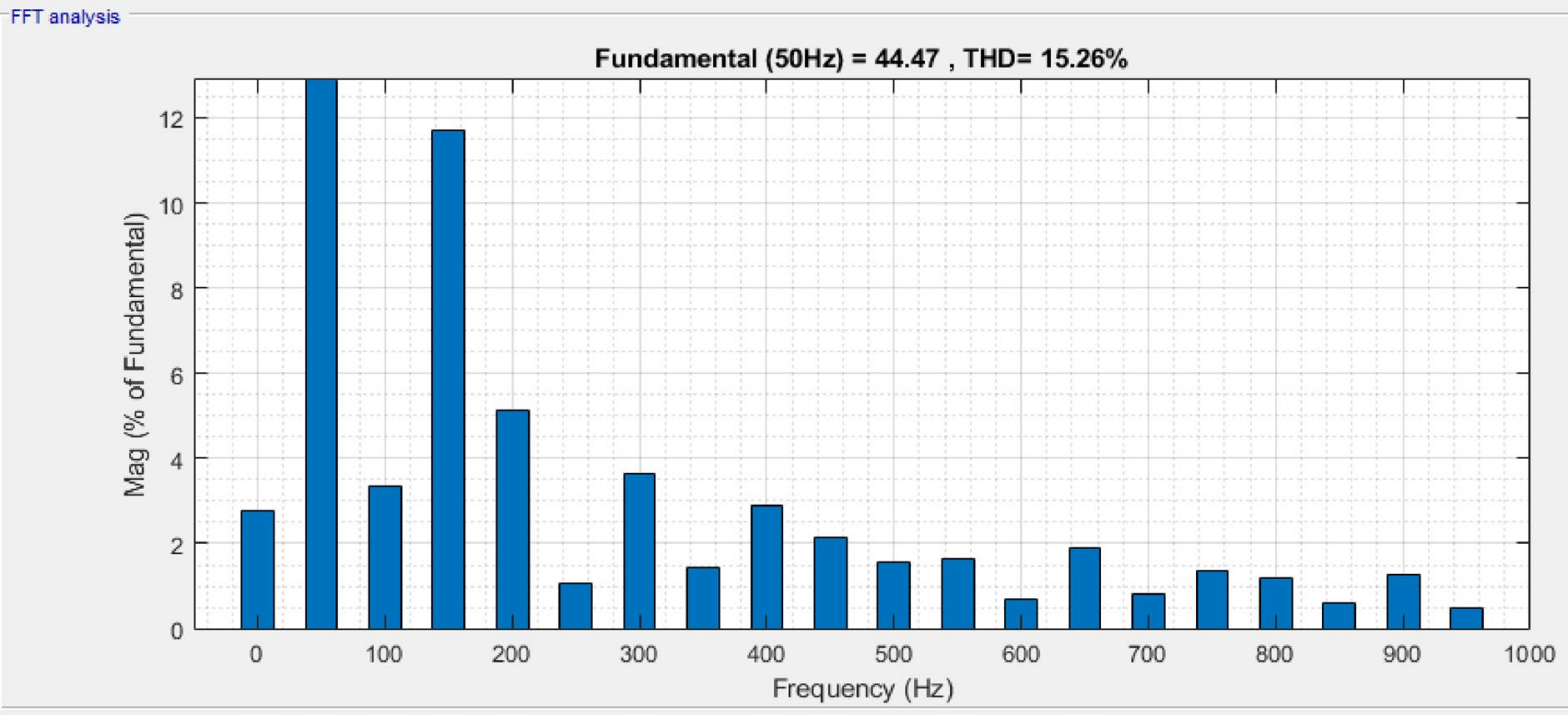
Frequency band for load voltage of UPFC system.

The sequence converter generates a voltage, which is transmitted to Ac terminal by a series linked transformer, thereby generating an injected AC voltage signal. Figure 8 shows a frequency spectrum of the load current with THD equal to 6.06% and Fig. 9 shows the corresponding load voltage spectrum, THD = 15.26%. Under such a condition, active and reactive power of UPFC is 0.29 MW and 0.063MVAR respectively which tally with the observed load current THD.

### Simulation result of DPFC system

**Fig. 10:**
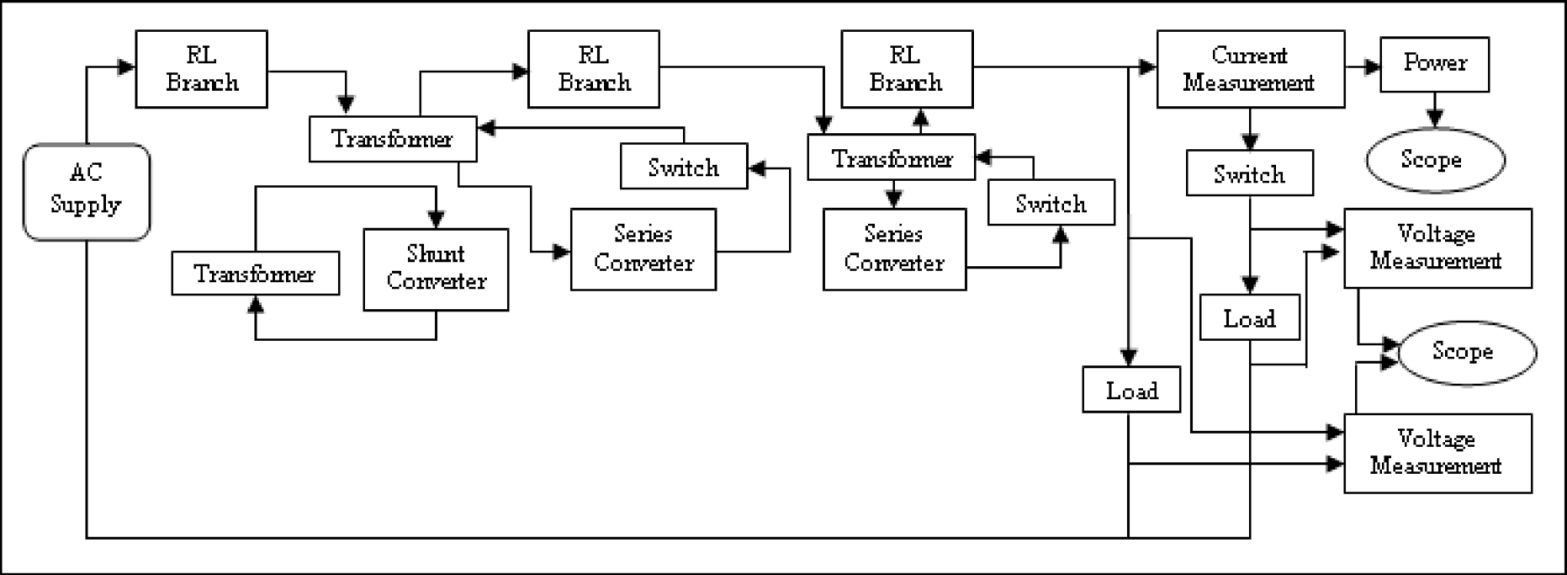
Line model of dual bus structure of DPFC system.

**Fig. 11:**
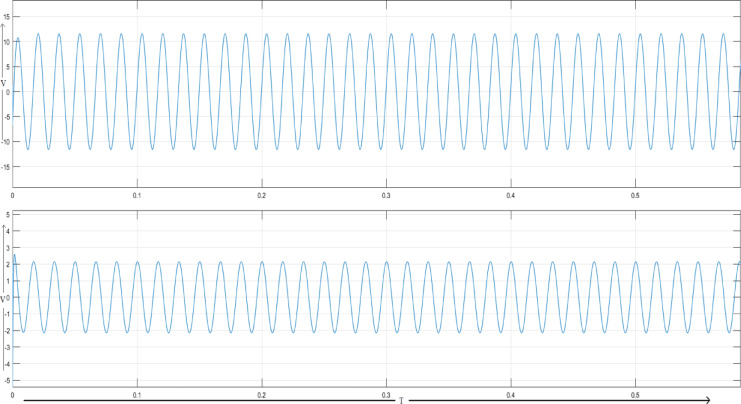
Voltage across load 1 and load 2 for DPFC system.

Figure 10 illustrates MATLAB/Simulink model of a dual‑bus system with a DPFC. Voltage injection is done with two series converters. Figure 11 shows Voltages across Load-1 and Load-2. A new load is introduced at time 0.2 s, with the first series converter being turned on at time 0.3 s and the second series converter activated at time 0.6 s.

**Fig. 12:**
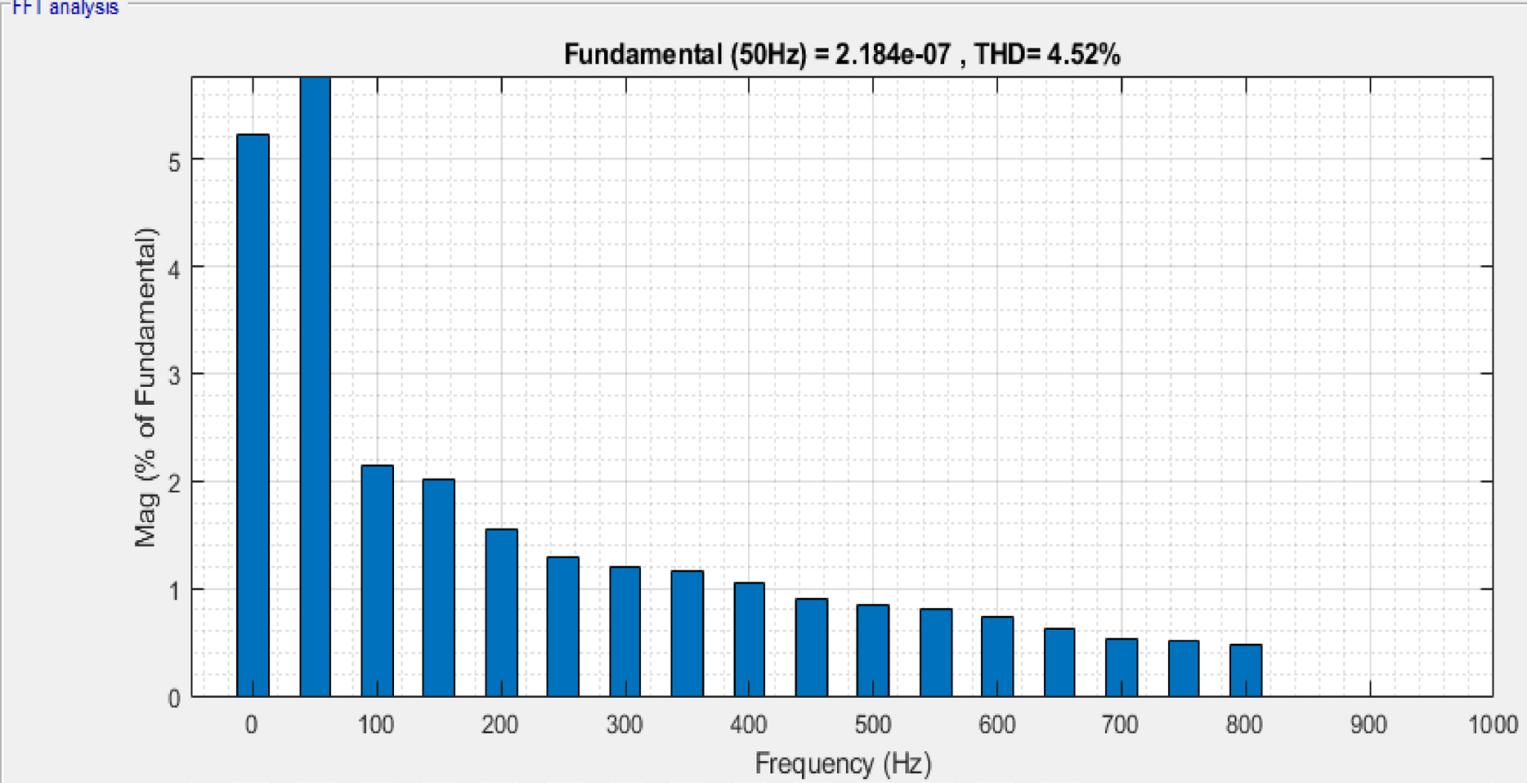
Frequency band for load current of DPFC system.

**Fig. 13:**
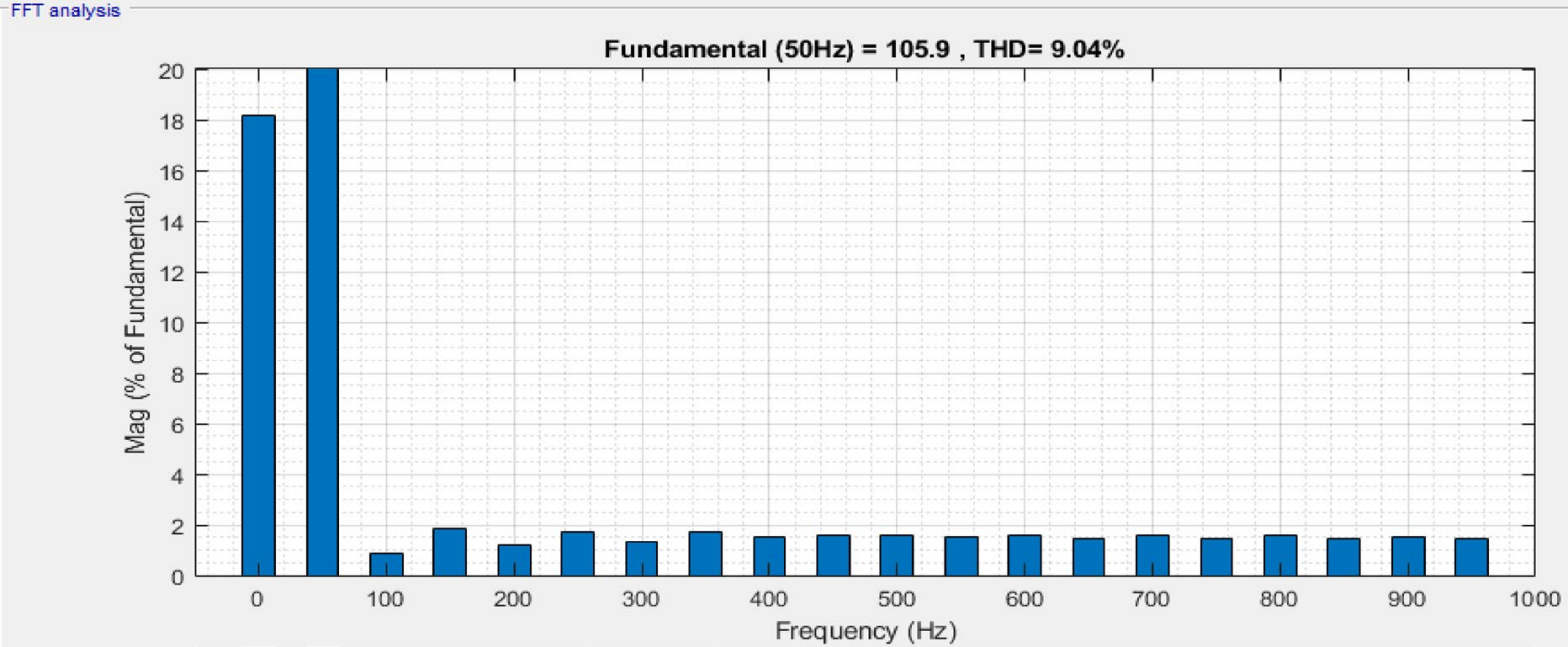
Frequency band for load voltage of DPFC system.

The active and reactive powers preserved are 0.30 MW and 0.079MVAR for DPFC, which shows a substantial improvement in power transfer for the proposed configurations than the conventional configurations. From the load current frequency spectrum in Fig. 12, the THD is 4.52%, while the load voltage spectrum Fig. 13 has a THD of 9.04%. The total active power rises, meaning that more useful work is delivered, and the reactive power increases to improve voltage regulation and ultimately system stability. While reactive power does not perform any useful work, its management will decrease the losses on transmission lines and increase the system efficiency. It is critical for keeping voltage levels throughout the grid steady and preventing drops or surges that can damage equipment.

In Table 2, comparison of voltage, real power, reactive power and THD in both systems is evaluated. A relative examination of DPFC and UPFC shows that the former provides better receiving‑end voltage, increased real and reactive power transfer, as well as lower current and voltage THD. This shows that DPFC has better overall power quality improvement than UPFC.

**Table 2:** Key performance aspects: power quality and harmonics.

FACTS device	Voltage (V)	Real power (MW)	Reactive power (MVAR)	Voltage THD	Current THD
UPFC	1100	0.290	0.063	15.26%	6.06%
DPFC	1100	0.301	0.079	9.04%	4.52%

**Fig. 14:**
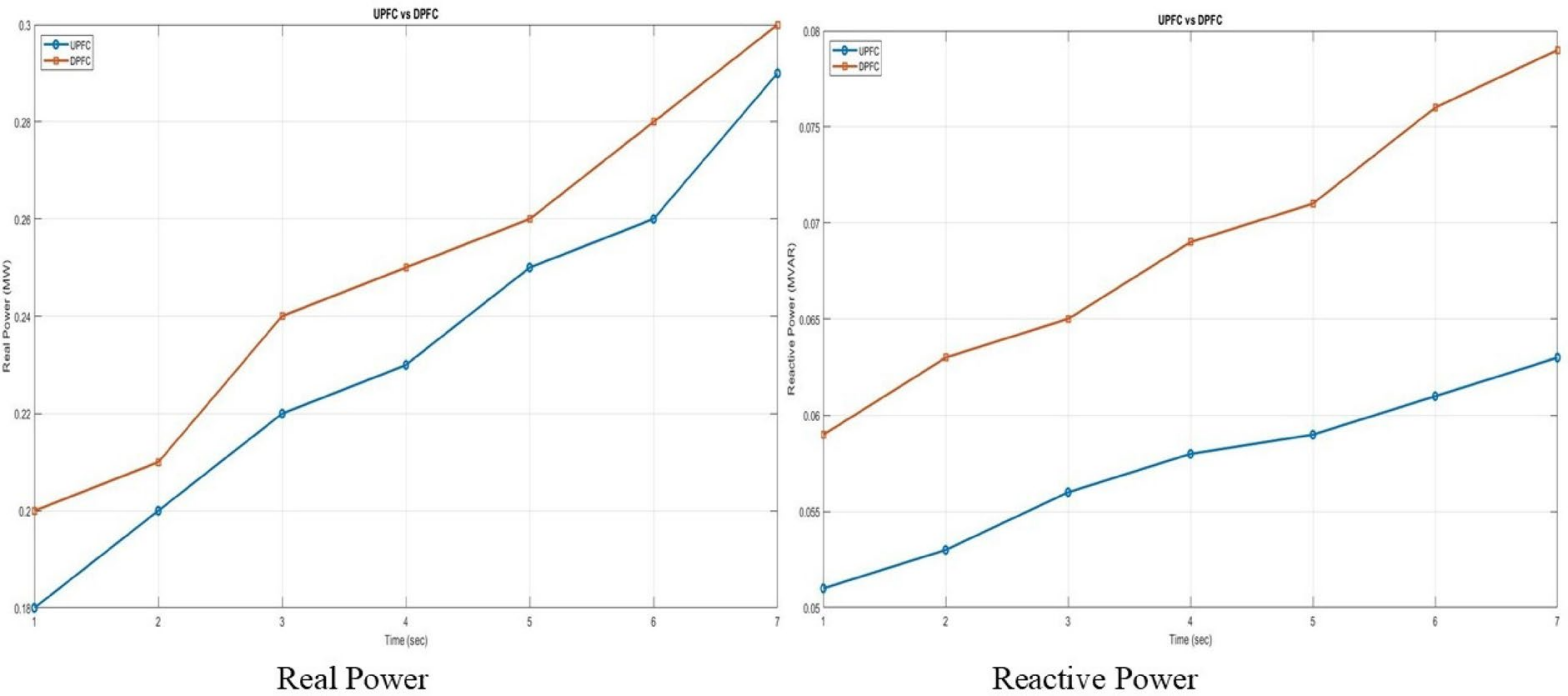
Real power and reactive power comparison of UPFC and DPFC.

A graphical comparison of active and reactive power for UPFC and DPFC is depicted in Fig. 14. Unlike the UPFC, both devices enhance reactive power support with time, however at every time interval observed, reactive power compensation is highest when using the DPFC.

**Fig. 15:**
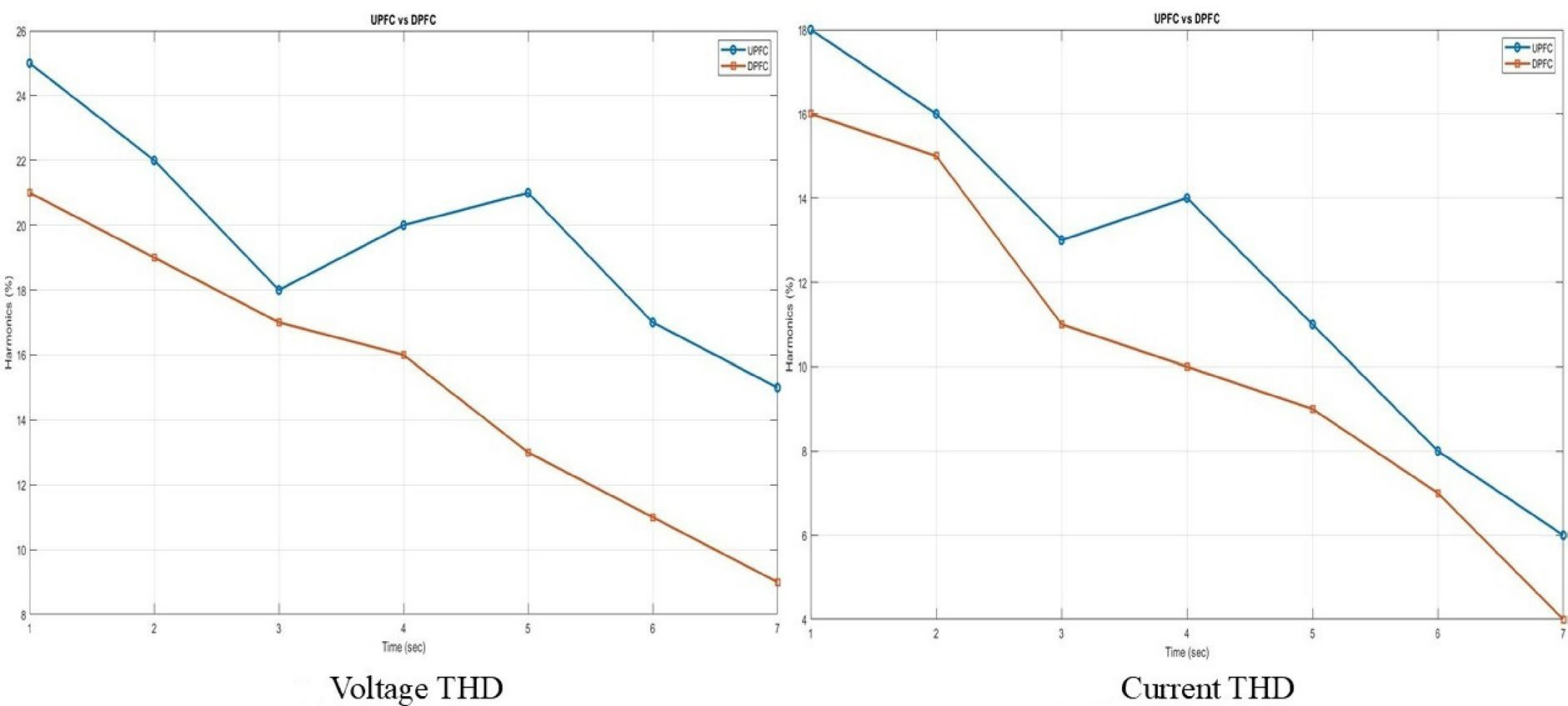
Voltage THD and current THD comparison of UPFC and DPFC.

The comparison of voltage and current harmonics for two devices is given in the graphical diagram shown in Fig. 15. The results indicate that the UPFC and DPFC reduce the voltage and current THD with each passing time step, which enhances power quality. On the other hand, the DPFC outperforms UPFC in both groups consistently showing lower rates of THD proving to be a better candidate for harmonic mitigation.

### Fault condition analysis

In order to check the performance of DPFC under a fault condition, a three‑phase fault is applied at 0.2 s and cleared at 0.3 s on the transmission line. The voltage recovery, power stabilization and harmonic distortion compensation performance of the DPFC is analysed.

**Fig. 16:**
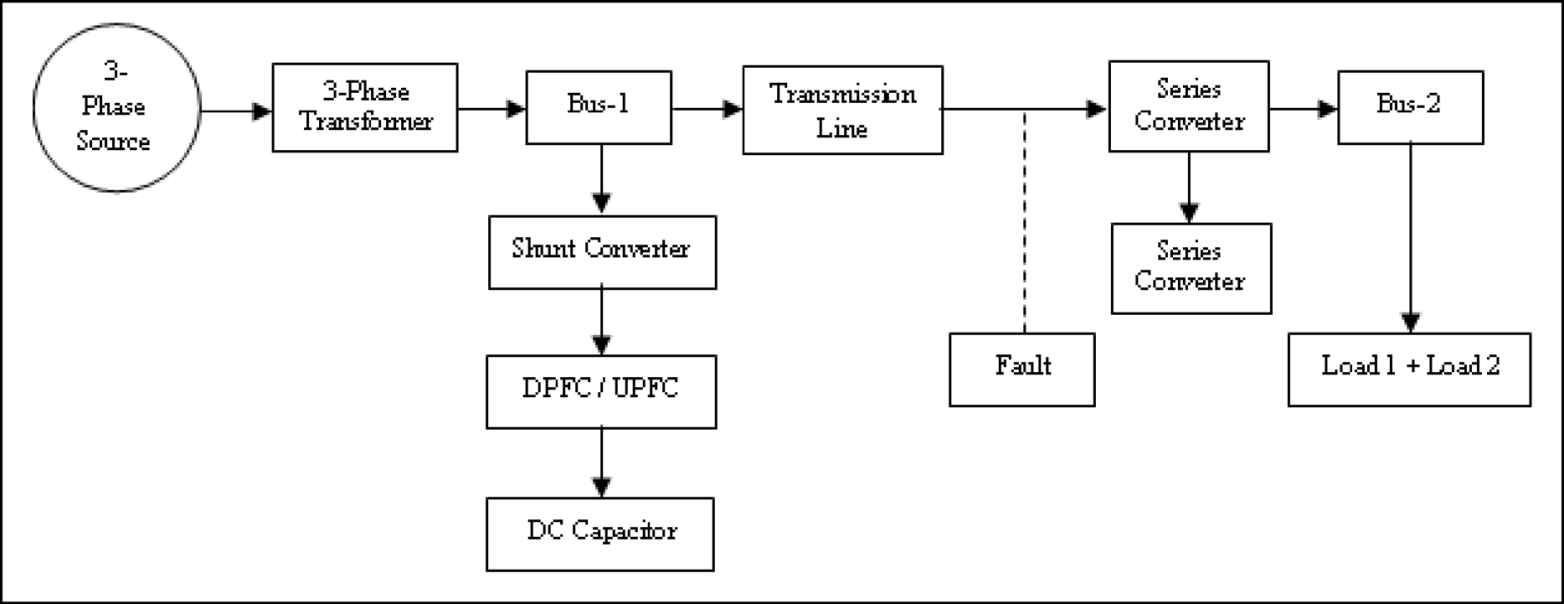
Line model of dual bus structure of UPFC/DPFC system with fault in transmission line.

**Fig. 17:**
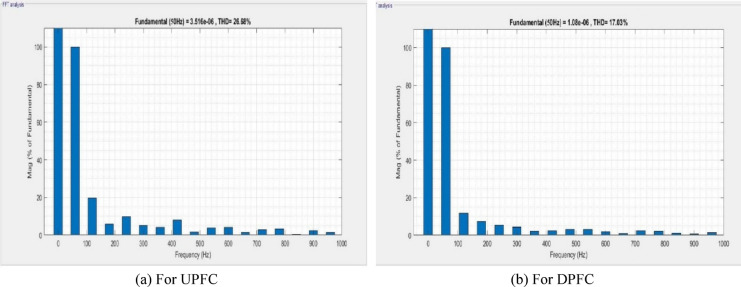
Frequency band for fault voltage of UPFC and DPFC system.

**Fig. 18:**
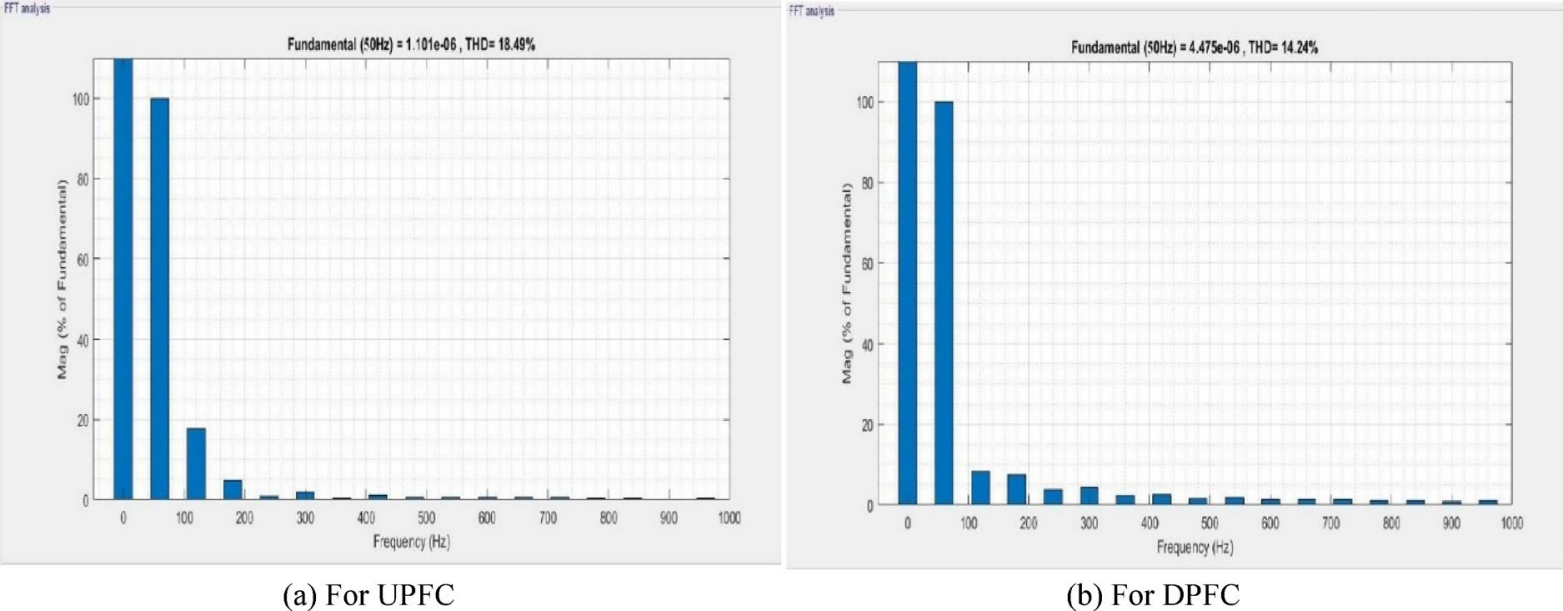
Frequency band for fault current of UPFC and DPFC system.

A MATLAB/Simulink model of the dual‑bus system by means of fault in transmission line is shown in Fig. 16. The UPFC or the DPFC is connected to the system for comparison. Essentially, the UPFC continues to provide 0.250 MW of active power and 0.058MVAR of reactive power in fault condition. The UPS is castoff to solve load voltage frequency spectrum for UPFC and DPFC, which can be seen in Fig. 17 with a voltage THD of 26.86% for the UPFC and a THD of 17.03% for the DPFC. As compared with the UPFC, the DPFC maintains its active and reactive powers at 0.280 MW and 0.072MVAR during fault. At this time, according to frequency spectrum of the load current in Fig. 18, the THDI was measured as 18.49% for UPFC and 14.24% for DPFC. An uncompensated system suffers a severe voltage sag during the fault condition. However, due to the DPFC compensation, the voltage profile gradually returns back to its previous value. Also, it’s shown in DPFC stabilizes active and reactive power over a shorter time giving it improved dynamic response. Additionally, THD levels are considerably lower for the DPFC system compared with those of UPFC‑based system which substantiates the better disturbance rejection capability of DPFC under fault conditions.

In Table 3, Evaluation of voltage, real power, reactive power and THD during the three‑phase fault condition is discussed. Comparatively, the DPFC system exhibits improved receiving‑end voltage, better real and reactive power transfer ability, as well as reduced total harmonic distortion (THD) in both current and voltage compared to the UPFC system. During fault conditions these results demonstrate that DPFC has improved performance than UPFC as far as power quality is concerned.

**Table 3:** Key performance aspects during fault condition: power quality and harmonics.

FACTS device	Voltage (V)	Real power (MW)	Reactive power (MVAR)	Voltage THD	Current THD
UPFC	1100	0.250	0.058	26.68%	18.49%
DPFC	1100	0.280	0.072	17.03%	14.24%

### Voltage profile under load variation

**Fig. 19:**
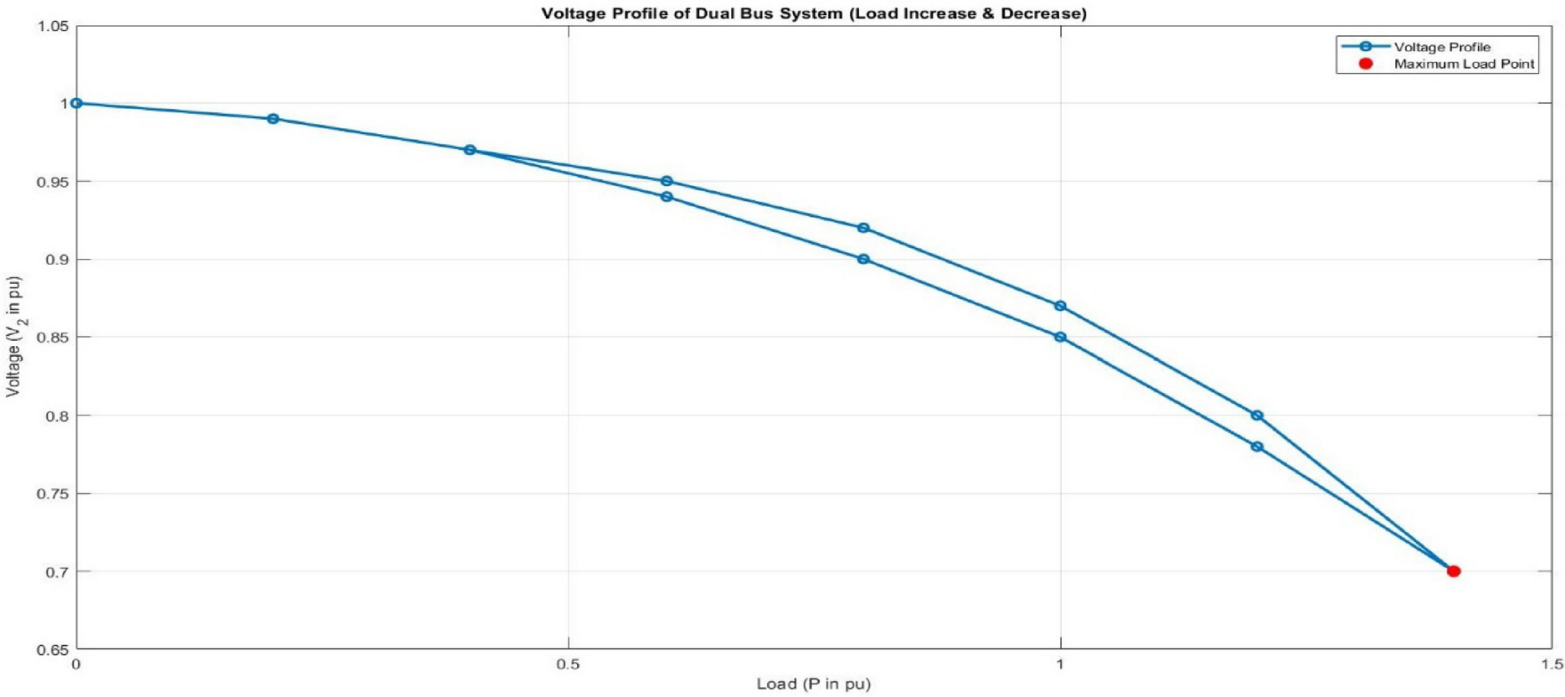
Voltage profile of dual-bus system under load variation.

The voltage profile of dual-bus system is shown in Fig. 19 during load variation. With increment in load, voltage deviations and reactive power demand of system also increases. As a result, with increased load the bus voltage drops gradually, depicting conventional voltage drop based behaviour under dual-bus configurations. Past a certain load level, the voltage drop becomes sharper, indicating the danger of system instability. Voltage regulation and load management is critical to confirm stability of system. The DPFC performs these functions by dynamically compensating for state deviations through reactive power compensation, subsequently ensuring the stability of voltage. Compared to UPFC, the DPFC can response itself and recover the voltage more quickly.

Table 4 assessment is discussed for UPFC and DPFC when subjected to load change and fault conditions displays superior performance of DPFC in terms of voltage stability, recovery time, and power quality. Despite variations in loading, UPFC is subjected to a sudden drop of voltage along with slower recovery and high THD rate contrary to DPFC which maintains low voltage decay, rapid current recovery and reduced THD. In the same manner, under fault condition, UPFC exhibits significant voltage drop with reasonable recovery and higher harmonics distortion but DPFC apparently reduces the voltage dip, restores system almost immediately while maintaining lower THD levels. In general, DPFC has more effective and steady performance because of decentralized construction and improved control.

**Table 4:** Dynamic condition performance.

Condition	Voltage dip	Recovery time	THD (%)
UPFC (load change)	High	Slow	Higher
DPFC (load change)	Low	Fast	Lower
UPFC (fault)	Severe	Moderate	High
DPFC (fault)	Reduced	Fast	Low

## Conclusion

Its aim is to provide a detailed comparative evaluation between Unified Power Flow Controller (UPFC) and its recent extension, Distributed Power Flow controller (DPFC), as an alternative way of directing power flow in transmission systems and creating voltage stability and adequate conditions. A maturing capacity market driven by the increasing demand of electrical energy and the challenge of transmission infrastructure expansion prompt attention to FACTS‑based devices. It was found from simulation results through MATLAB/Simulink that both UPFC and DPFC were capable of controlling active and reactive power in addition to improving voltage profiles in dynamic operating condition (key simulation parameters are summarized in Table 5), while it has capability for disturbance mitigation. In contrast, DPFC outperforms in all measured aspects. Also, under normal operating conditions with respect to active power transfer (i.e., 0.301 MW versus 0.290 MW), reactive power support (i.e., 0.079MVAR versus 0.063MVAR), and total harmonic distortion (viz., voltage THD: 9.04% vs. 15.26%; current THD: 4.52% vs. 6.06%), the performance of DPFC is significantly better than that of SSSC clone device. Under three‑phase fault, it has been shown that the dynamic response of DPFC is better than that of UPFC; providing active power 0.280 MW and reactive power 0.072MVAR with voltage THD (i.e., 17.03% vs. 26.68%) and current THD (i.e., 14.24% vs. 18.49%) comparatively lower than UPFC. Furthermore, the DPFC demonstrates faster voltage recovery and enhanced fault tolerance. Notably, the removal of a common DC link in DPFC, along with the application of dispersed single‑phase series converters, provides reduced cost and improved reliability and operational flexibility. Also, it is more appropriate for contemporary and complicated power systems because of its performance-oriented system fault retention and speedy voltage recovery. The DPFC is a superior option for UPFC in terms of functionality, reliability and cost-effectiveness but even with high performance under dynamic and disturbed conditions. Thus, it provides a potential solution for smart grid and advance transmission system applications in the near future.

**Table 5:** Key parameters related simulation of UPFC and DPFC device.

Parameters	UPFC	DPFC
System test voltage	1100 kV	1100 kV
Base power $\left({S}_{base}\right)$	100 MVA	100 MVA
Base voltage $\left({V}_{base}\right)$	230 kV	230 kV
Frequency	50 Hz	50 Hz
Transmission line	Resistance $\left(R\right)$= 0.02 p.u	Resistance $\left(R\right)$= 0.02 p.u
	Reactance $\left(X\right)$= 0.25 p.u	Reactance $\left(X\right)$= 0.25 p.u
Series converter rating	100 MVA	100 MVA
Shunt converter rating	100 MVA	100 MVA
Series converter	Injected voltage $\left({V}_{se\_i}\right)$ = 0.1 p.u	Injected voltage $\left({V}_{se\_i}\right)$ = 0.02 p.u
	Transformer per module: ${R}_{se}$ = 0.01 p.u ${X}_{se}$ = 0.1 p.u	Transformer per module: ${R}_{se}$ = 0.005 p.u ${X}_{se}$ = 0.05 p.u
Shunt converter	Voltage $\left({V}_{sh}\right)$ = 1.0 p.u	Voltage $\left({V}_{sh}\right)$ = 1.0 p.u
	Transformer: ${R}_{sh}$ = 0.01 p.u ${X}_{sh}$ = 0.08 p.u	Transformer: ${R}_{sh}$ = 0.005 p.u ${X}_{sh}$ = 0.06 p.u
PWM control	Modulation index = 0.9	Modulation index = 0.85
PI controller gains	DC voltage control: ${K}_{p}$ = 0.6 ${K}_{i}$ = 50	Power flow controller: ${K}_{p}$ = 0.45 ${K}_{i}$ = 30
	Voltage control: ${K}_{p}$ = 0.5 ${K}_{i}$ = 40	Voltage controller: ${K}_{p}$ = 0.4 ${K}_{i}$ = 35

## Data Availability

The data obtained through the experiments are available upon request from corresponding author.
